# Transcutaneous Tibial Nerve Stimulation for Primary Dysmenorrhea: A Protocol for a Randomized Controlled Trial

**DOI:** 10.3390/healthcare11111633

**Published:** 2023-06-02

**Authors:** Marta Correyero-León, Rocío Llamas-Ramos, Javier Calvo-Rodrigo, Jorge Juan Alvarado-Omenat, Inés Llamas-Ramos

**Affiliations:** 1 CEIP Simón de Colonia, Calle Padre Janáriz, 11, 09400 Aranda de Duero, Spain; marta.correyero@hotmail.com; 2 Department of Nursing and Physiotherapy, Faculty of Nursing and Physiotherapy, Universidad de Salamanca, Avda, Donantes de Sangre s/n, 37007 Salamanca, Spain; 3 CEE Fuenteminaya, Calle Padre Janáriz, 11, 09400 Aranda de Duero, Spain; 4 FisioSport Salamanca, 12 de Octubre, n° 2, 37008 Salamanca, Spain; 5 University Hospital of Salamanca, P.° de San Vicente, 182, 37007 Salamanca, Spain

**Keywords:** primary dysmenorrhea, posterior tibial nerve stimulation, pain, physiotherapy, protocol

## Abstract

Primary dysmenorrhea (PD) is a painful menstruation that can persist for the duration of a woman’s fertile life. Non-steroidal anti-inflammatory drugs, hormonal therapy, physiotherapy techniques, etc., are the main treatments. The main objective of this study is to evaluate the effectiveness of transcutaneous posterior tibial nerve stimulation (TTNS) in PD patients. The study will consist of a single-blind randomized clinical trial, parallel-assigned with two arms. Women with PD (18–43 years) with regular menstrual cycles and at least 4 points in VAS will be randomly divided into experimental (TTNS) and placebo (simulated stimulation) groups during 12 treatment sessions (1 session/week) and several follow-ups: monthly during treatment and 1, 3 and 6 months after. Maximum and mean pain intensity, pain duration, pain severity, number of anti-inflammatory drugs, quality of life, sleep quality, overall improvement, treatment satisfaction and secondary effects will be measured once a month every 6 months and at 3 and 6 months. The Student’s *t*-test for independent samples or the Mann–Whitney U test will be used. The literature shows effective physiotherapeutic techniques for PD in the short term, which do not act on causes and have limitations. The TTNS technique can be used in transcutaneous and percutaneous modalities, with similar effectiveness, but the transcutaneous causes less discomfort. TTNS modulates pain, and long-term benefits could be achieved at low cost and without patient discomfort.

## 1. Introduction

Primary dysmenorrhea (PD) is a painful menstruation that usually begins six to twelve months after menarche and can remain throughout the fertile life of women, and is generally associated with a normal ovulatory cycle and without pelvic pathology [1,2,3,4].

PD affects approximately between 45–95% of women; however, a high prevalence has been found in adolescents and young women (16–25 years old) [1,3,5]. It is estimated that approximately 10–25% of women will suffer very severe pain that prevents them from carrying out daily activities [2,6,7].

The current PD pathogenesis is mainly related to an excessive release of prostaglandins by the endometrium into menstrual fluid. Prostaglandin F2 alpha (PGF2α) and natural prostaglandin E2 (PGE2) induce vasoconstriction and contractions of the myometrium. High levels cause uterine hypercontractility that triggers hypoxia and endometrial mucosa ischemia. This uterine hypercontractility together with hypoxia and ischemia could be the cause of the cramp-like pain in PD. It also has associated systemic symptoms with vasopressin (uterine flow reduction), local factors (cervix stenosis) and, sometimes, psychological factors [2,3,6,8].

Primary dysmenorrhea is commonly described as cramping (cramping or spasmodic pain) or aching or dull aching (pain that is not severe but is insidious or continuous and generally difficult to describe or locate). The pain is commonly localized on the lower abdominal or suprapubic region, with or without radiation to the lower back, medial thigh or upper legs. It can start a few hours before or after menstrual bleeding onset, lasts approximately 48–72 h, and is most severe during the first or second day of menstruation [2,3,4,5,6,7,8]. In 50% of cases, women may suffer other associated symptoms like nausea, dizziness, vomiting, diarrhea, fatigue, headache, appetite loss, edema and a feeling of heaviness, insomnia, negative psychological effects and lipothymia due to hypotension [1,2,3,9]. In many cases, this situation causes school and work absenteeism and limits social, academic and sports activities [1,3,8,10]. Dysmenorrheic pain represents an immediate negative impact on quality of life, mood and sleep during menstruation compared to the pain-free follicular phase of women with PD and compared to the menstruation phase of women without pain [2,6].

Pharmacological treatment is based on non-steroidal anti-inflammatory drugs (NSAIDs) and hormonal regulation (oral contraception) [1,3]. These treatments are considered quite effective, but have negative effects (gastrointestinal, renal and cardiovascular) not tolerated by all patients [3,11,12].

In addition, there are various physiotherapy treatments for PD pain which do not appear to have adverse effects and are usually well tolerated by patients; they include transcutaneous electrical nerve stimulation therapy (TENS) [13,14,15], manual therapy [16,17], acupuncture [18,19], cryotherapy [20] and therapeutic exercise [4,21,22,23], which has shown positive results [24].

In recent years, a novel technique has emerged that could be aimed at the treatment of uterine hypercontractility, in addition to reducing pain. This technique is called transcutaneous posterior tibial nerve stimulation (TTNS), which consists of a peripheral neuromodulation where the posterior tibial nerve is electrically stimulated above the medial malleolus. This technique was first described by McGuire et al. in 1983 for incontinence treatment using two transcutaneous electrodes, one on each leg [25]. In 1999, Stoller adjusted this method to the percutaneous technique and placed the two electrodes on the same leg [26,27]. Since then, the technique (both transcutaneously and percutaneously) has been studied for urological conditions (overactive bladder [28,29] and fecal incontinence [30,31]) and gynecological pathologies (chronic pelvic pain [32,33,34]), with effective results in reducing symptoms and quality of life improvement.

The technique action mechanism in urological dysfunctions could be the modulation of reflex pathways, rebalancing the inhibitory and excitatory impulses that control bladder function at the spinal cord level [29,32]. Different theories have been considered: the stimulation of somatic fibers modulates/inhibits A-delta and C-afferent fibers, which will reduce pain sensation (gate theory); or endorphins increase at the spinal level and c-fos expression decreases in the central nervous system [32,35].

There are no specific studies that treat PD using TTNS in the literature. Physiotherapists have tools for chronic pain management and health promotion, having skills to perform PD interventions [3,21].

The posterior tibial nerve (L4–S3) has the same medullary levels as the hypogastric sympathetic plexus (L4–L5) and the pelvic parasympathetic plexus (S2–S4). Its stimulation produces impulse rebalancing in these centers, as is the case with the posterior tibialis relationship with the voiding centers [36,37]. Through that stimulation, uterine myometrium contractions are reduced; pain is alleviated by the decrease in uterine hypercontractility. This reduction in pain improves quality of life and sleep.

The main objective of this study is to verify whether posterior tibial nerve transcutaneous stimulation improves pain, quality of life and sleep deficiency in patients with PD in the short, medium and long term.

## 2. Materials and Methods

### 2.1. Objectives

#### 2.1.1. Main Objective

- To prove the Effectiveness of TTNS Treatment for PD in the Short, Medium, and Long Term

#### 2.1.2. The Secondary Objectives Are

- To evaluate TTNS effectiveness in reducing NSAIDs in the short term.
- To evaluate TTNS effectiveness in reducing pain in the short term.
- To evaluate TTNS effectiveness in improving the quality of life of women with PD in the short term.
- To evaluate TTNS effectiveness in improving sleep deficiency among women with PD in the short term.
- To confirm if any decrease in pain is maintained over time.
- To confirm if any improvement in quality of life is maintained over time.
- To confirm if any improvement in sleep deficiency is maintained over time.
- To evaluate patient satisfaction with the treatment.
- To explore adverse reactions arising from the treatment.

### 2.2. Hypothesis

#### 2.2.1. The General Hypothesis Is That

- The Application of TTNS Results in Decreased Pain during Menstruation, and An Improvement in Quality of Life and Sleep Deficiency

#### 2.2.2. The Secondary Hypotheses Are That

- TTNS influences the nerve centers producing a modulation of reflex pathways, thus rebalancing the inhibitory and excitatory impulses that control painful uterine afferents.
- TTNS modulates A-delta and C-afferent fibers, which reduces pain sensation in the short-medium term and is maintained 6 months after treatment.

### 2.3. Design and Setting

This is an experimental study, a single-blind randomized clinical trial with a parallel assignment study with 2 arms, which will be implemented in a physiotherapy center.

The study will consist of four parts:

First interview. The study characteristics will be explained to the participants in a 30-minute interview. A signed informed consent document and a filled-in medical history sheet will be required to participate. This medical history sheet will be filled out by the participant with the support of the researcher during this first interview. It will consist of the following sections: demographic characteristics, life habits, general medical history, gynecological history, obstetric history, menstrual history and history of menstrual pain.

Evaluation phase. Several questionnaires during two consecutive menstrual periods will be filled out. These questionnaires will be given to the participants at the first inter-view and they will have to self-administer them during the following two menstrual periods. The objective of this phase will be to know the baseline status of the participants with respect to the variables studied.

Intervention phase. Participants included in the study will be randomly assigned to two groups, an interventional and a control group, at a 1:1 ratio. Participants will not know which group they belong to. One session a week (12–30 min) will be conducted. Both groups will receive a symmetrical biphasic current in different leg locations. The stimulation will be performed transcutaneously through two/four electrodes at the leg. The technique shall be always painless, without harmful effects. Patients must fill in the questionnaires and on the last day of the treatment a treatment satisfaction scale will be requested. These questionnaires will be self-administered. The satisfaction and global improvement scales will be completed on the last day of treatment in the office, but on an individual basis, without the advice of the researcher. In addition, after each treatment session, participants will fill out a side-effect questionnaire.

Re-assessment phase. The same questionnaires will be filled in a month after treatment, and 3 and 6 months after the treatment ends. In addition, at the end of the 6-month follow-up period, patients will be asked to fill out the adverse effects questionnaire and the satisfaction and global improvement scales again. All these questionnaires will be self-administered.

### 2.4. Sample

**Figure 1:**
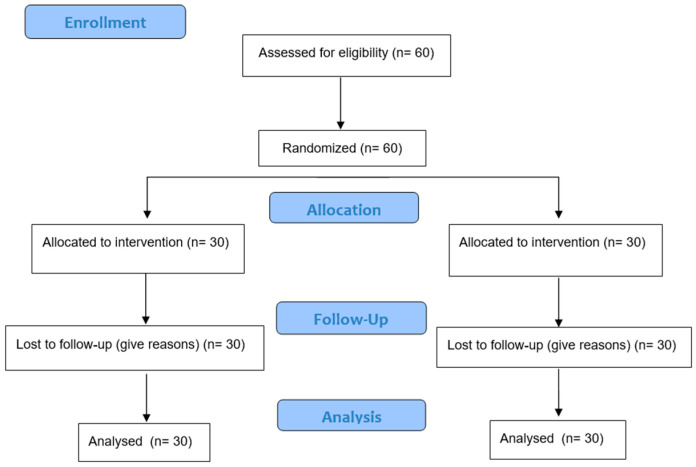
CONSORT flow diagram.

It is estimated that the study will have a sample of 60 participants (Figure 1). 

The inclusion criteria for participant selection will be the following: women aged between 18 and 43 years, who have regular menstrual cycles (21–35 days) and who present between 4–10 points of the visual analogue scale (VAS) during half of their menstrual cycles per year and/or during their last three cycles for pain located at the suprapubic area, abdomen, lower lumbar area, perineum and/or medial side of the thighs in the first two days of their menstrual cycle.

The exclusion criteria established will be: women with an intrauterine device implanted or women with hormonal treatment; those diagnosed in the last 18 months of secondary dysmenorrhea by their gynecologist (endometriosis, ovarian cyst, etc.); abdominal or pelvic surgeries during the study or having given a birth in the last 6 months; presence of skin lesions such as scars, erosions, etc., on the upper-internal face of the ankles; pregnancies; and women who have pacemakers, uncorrected coagulopathies, severe comorbid disorder, cancer (in the last 5 years or currently), severe mental disorders or lower-extremity neuropathies and physiotherapy treatments for this pathology one month before the study onset.

### 2.5. Variables

The following variables will be assessed during the study implementation.

#### 2.5.1. Pain

- Maximum and mean pain intensity and pain duration: The visual analogue scale (VAS) will be used. It consists of a horizontal line of 10 cm: on the left is the absence or lesser intensity of pain, with 0: “no pain” and on the right the highest intensity, with 10: “the worst pain imaginable”. The patient will be asked to mark on the line the point that indicates the pain intensity that she feels. To know the score, the researcher will measure the distance from “no pain” to the patient’s mark [38,39,40]. The pain classification will be taken as mild pain (1–3 cm), moderate pain (4–6 cm) and severe pain (7–10 cm). The patient should fill in the scale each day of her menstruation indicating the maximum level of pain that she has experienced during each day.
- Pain severity: The McGill Short Questionnaire (SF-MPQ^®^) will be used. The questionnaire has three parts: the pain rating index; a sensory subscale with 15 items that are classified on an intensity scale where 0 = none, 1 = mild, 2 = moderate and 3 = strong, which ranges from 0 to 45 points; a VAS (ranged from 0 to 10); and the current pain index (a subscale that measures pain intensity from 0 to 5). The higher the SF-MPQ^®^ score, the greater the pain severity [40,41]. The patient should fill in the questionnaire on the day of maximum pain of her menstruation.
- Number of anti-inflammatory drugs taken by the patients: a diary kept by the researcher will be used. The patients will record each NSAID in the diary, what type and amount of it they take during all the days of their menstruation and whether there has been pain relief with each dose.

Pain will be measured once a month every 6 months and at 3 and 6 months.

#### 2.5.2. Quality of Life

The SF-36v2^®^ Health Questionnaire will be used. It is made up of 35 items that measure 8 dimensions of health-related quality of life. In addition, it includes a change self-assessment item that is not used in scoring. The instrument items cover: physical function, physical role, bodily pain, general health, vitality, social function, emotional role and mental health. The higher the score on the SF-36, the better the health. The “acute” version (1 week) will be used [42,43]. The patient must fill in the questionnaire on the last day of her menstruation. Quality of life will be measured once a month every 6 months and at 3 and at 6 months.

#### 2.5.3. Sleep Quality

The Pittsburgh Sleep Quality Index (PSQI^®^) will be used. It is a self-administered questionnaire that consists of 19 items in addition to 5 questions for the bed partner. The latter are used as clinical information, but do not contribute to the total score of the index. The 19 items analyze different determining factors of sleep quality, grouped into 7 components: sleep quality, latency, duration, efficiency, disturbances, use of sleep medication and daytime dysfunction, where each component is scored from 0 to 3. The sum of the 7 components gives the total score of the PSQI^®^, which ranges between 0 and 21 points: the higher the score, the poorer the sleep quality [44,45]. Buysse et al. [44] propose a cut-off point of 5 (score ≥ 5 defines “bad sleepers”). The patient must fill in the questionnaire on the last day of her menstruation. Sleep quality will be measured once a month every 6 months and at 3 and at 6 months.

#### 2.5.4. Overall Improvement and Treatment Satisfaction

- Patient’s Global Impression of Change Questionnaire (PGIC). The questionnaire consists of 7 items. The patient must choose the one that most closely approximates the improvement obtained with the treatment. The higher the score, the worse the impression of change [46]. The patient should fill it in at the end of the treatment and at the end of the reevaluation. Overall improvement will be measured at 5 months and at 11 months.
- As a subjective measure of the success of the treatment, patients will be asked, using a Likert scale, if they would like to continue with the treatment to maintain the objectives achieved. The scale shows the degree of agreement or disagreement with the treatment. The patient should fill it in at the end of the treatment and at the end of the reassessment. It will be measured at 5 and at 11 months.

#### 2.5.5. Secondary Effects

Possible treatment adverse reactions will be collected, and patients will be asked to fill in a short questionnaire prepared by the investigator after each treatment session. Time frame: Once a week for 12 weeks during treatment and a follow up 11 months after the onset.

### 2.6. Procedures and Timing

The procedure of the study will be established in two arms.

#### 2.6.1. Arm 1, Experimental Group

TTNS group. Participants will receive an intervention session each week over a period of 12 weeks. The total application time will be 30 min. A symmetrical biphasic current will be applied, with a frequency of 20 Hz in continuous mode and an impulse duration of 200 μs. Participants will be placed in the supine position with the soles of the feet together and the knees flexed and abducted at 90°. Two adhesive electrodes will be used for each leg. The first will be 32 mm in diameter and placed on the posterior tibial nerve, 4–5 cm cranial to the internal malleolus, between the posterior edge of the medial border of the tibia and the soleus tendon. The second will be 50 mm × 50 mm and placed in the ipsilateral calcaneus. The electrodes will be connected to a NeuroTrac^®^ PelviTone stimulation device. The stimulation range will be selected according to the tolerable pain limit for the patient, between 1 and 30 mA (adjustable in 1 mA levels). An intensity elevation will be allowed each time the patient perceives the fading of the previous sensation due to accommodation. Under no circumstances should the stimulation cause a painful feeling.

#### 2.6.2. Arm 2, Control Group

One intervention session per week will be performed during a 12-week period time. The total application time will be 30 min. A discontinuous current at 2 Hz frequency and a pulsed frequency of 50 μs, with 2 s of work and 10 s of pause will be applied in other localization. The participant will be placed in the same position. Two 50 mm × 50 mm adhesive electrodes will be placed on the external face of the thigh, on a single leg. This area is outside the territory of the posterior tibial nerve. This simulated current is considered to be insufficient to achieve therapeutic effects in the body and to be outside of the usual ranges. The pain limit level of the patient will be considered for graduating the intensity (1–60 mA, adjustable in 1 mA levels). Patients will experience a low or moderate sensation and no muscle contraction will be achieved.

### 2.7. Data Collection and Management

The data will be collected through self-administered questionnaires. These data will be randomized using the SPSS v26 software (IBM Corporation, Armonk, NY, USA).

### 2.8. Data Analysis

For the statistical analysis, the corresponding statistical tests will be carried out to establish the differences between groups and differences within each group independently. 

The results will be expressed as mean ± standard deviation for the quantitative variables and as a number and percentage for the qualitative variables. Normality will be evaluated with the Kolmogorov–Smirnov test. The mean of the two groups will be compared using a Student’s *t*-test for independent samples or a Mann–Whitney U test as appropriate. Qualitative variables will be analyzed using an x^2^ test. The relationships of the quantitative variables to each other will be tested using the Pearson or Spearman correlation as appropriate. To analyze the effect of the intervention, the results obtained among the intervention group will be compared with those obtained among the control group. A value of *p* < 0.05 will be established as statistically significant.

### 2.9. Ethical Considerations

The study was approved on 12 December 2019, by the Ethics Committee for Drug Research, Valladolid East Health Area, of the Hospital Clínico Universitario de Valladolid, located at Hospital Clínico Universitario de Valladolid, Av. Ramón y Cajal, 3, 47003 Valladolid (Spain). The research will follow Helsinki and CONSORT guidelines and all participants will be informed and sign an informed consent to participate. The study has a ClinicalTrials.gov, accessed on 21 May 2023, registration number ID: NCT04896814.

## 3. Discussion

Dysmenorrhea is currently postulated as one of the health problems that most affect the quality of life of women, with repercussions on their working life. In addition, physiotherapy is very present today and treatments are emerging with the aim of improving pain and the quality of life of patients with urogynecological complications [31,32,46].

There are numerous studies in the literature about physiotherapy treatments which try to relieve menstrual pain [13,14,16,17,21,22]. There is considerable heterogeneity in the studies published related to physiotherapeutic treatments, with studies referring to manual therapy techniques, thermotherapy, cryotherapy, neuromuscular taping, therapeutic exercise and different electrotherapy techniques. Within the interventions related to electrotherapy, where the presented technique would be encompassed, the most used technique is TENS [13,14,47]. These studies compared the use of TENS for 3 months with a control group in a population of nulliparous and multiparous women of childbearing age, a sample similar to the presented study. Only one of the studies [47] did not allow the use of contraceptives and the other two studies allowed the use of NSAIDs. In the present study, women who have not used contraceptives or have an IUD implanted will be included, since in this case it is considered that it would be difficult to discriminate which effects could be attributed to the medication and which to the treatment applied in the study. On the other hand, the intake of NSAIDs is considered appropriate since it is taken as an indicator of pain improvement. As for the measurement tools, the vast majority of studies take the VAS as a reference for pain assessment, and statistically significant differences were observed when comparing between groups in the short-term decrease in pain [13,14,47]. Furthermore, in the study by Hai-Yan et al. [13], a statistically significant decrease in NSAID intake was observed when comparing both groups. On the other hand, two of the studies [13,14] did not show statistically significant improvements in the experimental group with respect to quality of life. Long-term follow-up is not observed in the studies presented, which is essential to understand the duration of the effects of the technique.

However, many limitations have been found in the reviewed publications, with low methodological quality. Likewise, it is observed that the treatment techniques used in these studies have been proposed to combat pain secondary to the underlying pathology and not to the cause itself, obtaining short-term results [8,13,21].

Posterior tibial nerve stimulation has been studied as a treatment for chronic pelvic pain with beneficial results in reducing pain and improving quality of life [32,33,34,35], although the literature analyzed is scarce and samples observed in studies are very small. 

In these studies, middle-aged men and women with a diagnosis of chronic non-cyclic pelvic pain of more than 6 months’ duration, in which PD was not found, were included. Although PD is considered a chronic pelvic pain, it is necessary to differentiate it from other non-cyclical pathologies such as endometriosis, interstitial cystitis, etc. Most of the intervention groups consisted of 12 weeks of treatment at the rate of one 30 min session per week of percutaneous application, inserting the needle 3–6 cm cephalad from the medial and posterior malleolus to the tibia bone and with a neutral electrode placed in the same leg in the plantar arch of the foot, by unilateral application. Stimulation parameters of 20 Hz frequency and 200 µs pulse duration and intensity at the motor contraction threshold were used. In the present study, the same application modality will be used, although it was decided to perform it bilaterally in order to increase the therapeutic effects and by transcutaneous application. The technique has been used over time in two variants, transcutaneous (using surface electrodes) and percutaneous (using needles). Most studies have used the percutaneous modality, but other studies have demonstrated similar transcutaneous modality effectiveness, with a discomfort reduction that patients feel when being stimulated [48,49]. All studies have suggested improvement in short-term pain, assessed with the VAS and/or the SF-MPQ. Regarding quality of life, some studies have assessed it using the SF-36 questionnaire, finding positive changes in some domains for the experimental group in the short term. However only one study [35] conducted long-term follow-up, which suggested that the technique is still effective 6 months after the end of its application, which would lead to an improvement of pain in the medium-long term. This is an interesting aspect of the present study, in which a long-term follow-up will be carried out up to 6 months post-treatment.

Finally, no studies addressing the treatment of PD using this technique have been found.

## 4. Limitations

The sample required to carry out the study requires very specific inclusion and exclusion criteria, which could limit the planned sample size.

Although it is not the aim of the present study there are no tools for measuring the rate of prostaglandins or uterine contractility.

## 5. Conclusions

The technique of transcutaneous stimulation of the posterior tibial nerve in its percutaneous modality could be considered as an alternative treatment for primary dysmenorrhea without adverse effects for women. This technique could be a useful, effective and safe tool for the treatment of women with primary dysmenorrhea improving pain, quality of life and sleep, and reducing work absenteeism.

## Data Availability

All data will be available under reasonable request to the corresponding author.
